# Supplementary material for: FlaHMM: unistrand flamenco-like piRNA cluster prediction in Drosophila species using hidden Markov models
Source: NAR Genom Bioinform. 2024 Sep 14;6(3):lqae119. doi: 10.1093/nargab/lqae119 (PMC11400887; doi:10.1093/nargab/lqae119)
Supplement: lqae119_Supplemental_Files [file lqae119_supplemental_files.zip › FlaHMM_supplement_240722.pdf]

Supplementary Information for

FlaHMM: unistrand *flamenco*-like piRNA cluster prediction in *Drosophila* species using hidden Markov models

Supplementary Methods
Methods S1: Centromere definition.

Supplementary Results
Results S1: Model performance depends on contig size.
Results S2: Model evaluation and class imbalance.

Supplementary Tables
Table S1: Overview of species included in the training and test sets.
Table S2: Definition of emissions.
Table S3: Performance on genome assemblies with *flam*-syntenic clusters.
Table S4: Performance on genome assemblies with *flam*-like clusters.
Table S5: Performance on genome assemblies with *flam*-syntenic clusters [5 kb bins, 0.075 threshold settings].
Table S6: Performance on genome assemblies with *flam*-like clusters [5 kb bins, 0.075 threshold settings].
Table S7: Detailed performance for top six model settings.
Table S8: proTRAC piRNA cluster predictions for *D. ficusphila*.

Supplementary Figures
Figure S1: Automatic annotation of centromeric regions.
Figure S2: Emission matrices depend on TE content threshold.
Figure S3: Overview of model performance by NX threshold and bin size.
Figure S4: Overview of model performance by threshold and bin size.
Figure S5: Additional details on model performance.
Figure S6: Model output for *D. oshimai*.
Figure S7: Model output for *D. innubila*.
Figure S8: Model output for *D. ficusphila*.
Figure S9: Overview of proTRAC predictions for *D. ficusphila*.

Supplementary References

Methods S1: Centromere definition

Centromeres in *D. melanogaster* are enriched for non-LTR retroelements belonging to the Jockey family, particularly G2/Jockey-3, although these elements are not exclusive to centromeres (1). Similar enrichment is observed in other species, such as *D. simulans*, where enrichment of G2/Jockey-3 elements at centromeres has been observed (2). This suggests a conserved role for retroelements in centromeric function, as proposed for other species (3).

While most transposon families, including LTR elements, are present in centromeres, distinguishing these regions from *flam*-like clusters is crucial. To achieve this, we annotated centromeric regions separately, allowing FlaHMM to discern them from *flam*-like regions. Genome assemblies with contigs labelled as the left and right arm of a chromosome (i.e., chr2L and chr2R, or chr3L and chr3R) were used in combination with predicted TE content to define centromeric regions. Each species' genome was binned into 100 kb bins and TE content was calculated per bin. A sliding window of 5 bins was used to evaluate TE content, with centromeric regions defined to start at the chromosome end and extend for as long as the mean TE content across the 5 bins was exceeding 20%. A visualisation of this calculation for *D. melanogaster* is shown in Figure S1, with metrics for all species available in the GitHub repository (centromere_definition, https://github.com/Hannon-lab/FlaHMM-supplement).

Results S1: Model performance depends on contig size

To systematically determine how performance depends on contig size, we evaluated the model on a subset of the full assemblies using N80, N85, N90, N95, N99, and N100 as contig cutoff (Figure S3). Notably, performance substantially improved when limiting evaluation to contigs corresponding to the 90th or higher most continuous percentile of the assembly, compared to using the full assembly (N100). We therefore concluded that most prediction errors occur at small and unplaced contigs, and although predictions can be made for all contigs, we recommend the exclusion of these when interpreting the results.

Results S2: Model evaluation and class imbalance

We note that representing and interpreting classification performance is challenging in situations with uneven class distribution, such as the one presented in our study. Since the class distribution was highly uneven (e.g. 87.6% None, 0.3% Cluster and 12.1% Centromere for *D. melanogaster*), we therefore represented model performance as the mean performance across all three classes throughout our study. However, it should be noted that performance varies substantially by class (Figure S5C), with larger classes obtaining higher performance, and that the global performance (weighted) is substantially better than the mean performance (Figure S5C).

Table S1: Overview of species included in the training and test sets. Annotated unistrand piRNA cluster coordinates were used as previously reported (4). Test refers to *flam*-syntenic clusters, whereas Test2 refers to *flam*-like ones without synteny.

			**Unistrand piRNA cluster**				
**Dataset**	**Species**	**Assembly**	**Name**	**Contig**	**Start**	**End**	**Strand**
Training	Dyak	GCF_016746365	*flam*	chrX	21,210,985	22,624,858	+
Training	Dsan	GCF_016746245	*flam*	chrX	21,033,264	21,845,911	+
Training	Dsim	GCF_016746395	*flam*	chrX	21,073,454	21,511,484	+
Training	Dmau	GCF_004382145	*flam*	chrX	21,407,318	22,252,287	+
Training	Dmel	dm6	*flam*	chrX	21,631,904	22,434,871	+
Training	Dsubp	GCF_014743375	*flam*	chrX	1,890,097	2,975,514	-
Test	Dbia	GCF_018148935	*flam*-syntenic	chrUn_025319364	24,741,581	24,969,825	+
Test	Dbia	d101g	*flam*-syntenic	contig_275	24,743,392	24,969,643	+
Test	Dbia	d15genomes	*flam*-syntenic	utg000088l	779,356	972,521	-
Test	Dere	GCF_003286155	*flam*-syntenic	chrUn_020825209	15,529,294	15,801,676	+
Test	Dere	d101g	*flam*-syntenic	contig_636	419,529	994,684	-
Test	Dere	droEre1	*flam*-syntenic	scaffold_4690	17,965,809	18,220,474	+
Test	Dere	d15genomes	*flam*-syntenic	utg000005l	2,667,515	2,942,695	+
Test	Dsuz	GCF_013340165	*flam*-syntenic	chrX_023496844	665,099	1,345,189	+
Test	Dtei	GCF_016746235	*flam*-syntenic	chrX	20,998,347	21,767,495	+
Test	Dtei	d101g_2733	*flam*-syntenic	contig_529	1	180,271	-
Test	Dtei	d101g_CT02	*flam*-syntenic	contig_903	563,752	778,755	-
Test2	Dfic	GCF_018152265	*flamlike1*	chrUn_025064569	438,589	994,838	-
Test2	Dfic	d101g	*flamlike1*	contig_636	419,529	994,684	-
Test2	Dfic	GCF_000220665	*flamlike1*	chrUn_016073254	1	77,405	-
Test2	Dosh	d101g	*flamlike2*	contig_1	23,542,221	24,075,495	+
Test2	Dath	GCA_008121215	*flamlike3*	chrX	35,768,372	36,552,428	+
Test2	Dazt	GCA_005876895	*flamlike3*	VCKU01000012	4,140,196	4,501,485	+
Test2	Dmir	GCF_003369915	*flamlike3*	chrXR	30,822,007	31,271,755	-
Test2	Dper	GCF_003286085	*flamlike3*	chrUn_020825317	2,171,578	2,418,435	+
Test2	Dper	d101g	*flamlike3*	contig_44	385,239	505,116	+
Test2	Dper	d15genomes	*flamlike3*	utg000136l	407,796	438,716	+
Test2	Dpse	d15genomes	*flamlike3*	utg000003l	435,828	675,327	+
Test2	Dpse	GCF_009870125	*flamlike3*	chrX	37,962,257	38,275,576	+
Test2	Dinn	GCF_004354385	*flamlike4*	chrX	30,916,784	31,158,180	-
Test2	Damb	d101g	*flamlike5*	contig_109	126,545	295,508	-
Test2	Dbif	GCA_009664405	*flamlike5*	chrA	13,681,722	13,800,479	-
Test2	Dobs	d101g	*flamlike5*	contig_185	1	66,608	-
Test2	Dobs	GCF_018151105	*flamlike5*	chrUn_024542773	1	56,444	-
Test2	Dtris	d101g	*flamlike5*	contig_125	2,204,925	2,260,492	+

Table S2: Definition of emissions. Emissions were defined based on three conditions relative to a threshold (τ) as specified below. ***G+*** and ***G-*** represent the Gypsy-family TE content (G) on the plus (+) and minus (-) strand, respectively, represented as a fraction of the total bin size.

LTR/Gypsy content			Emission	
$G+ - G- > \tau$	$G- - G+ > \tau$	$G+ + G- > \tau$	Plus strand	Minus strand
FALSE	FALSE	FALSE	0 (none)	0 (none)
FALSE	FALSE	TRUE	2 (both)	2 (both)
FALSE	TRUE		1 (one)	0 (none)
TRUE	FALSE		0 (none)	1 (one)

Table S3: Performance on genome assemblies with *flam*-syntenic clusters. Performance for the 5 kb bin, 0.025 threshold settings is shown as TPR and FPR, respectively. Species are sorted alphabetically and multiple assemblies for the same species are sorted based on N50 as an indication of assembly quality. Results shown for N90 contigs unless otherwise specified. Detailed metrics are shown in Table S7.

**Assembly**	**Cluster**	**TPR**	**FPR**	**Contigs**	**N50**	**Total size**
Dbia.GCF_018148935	*flam*-syntenic	1.0000	0.0177	283	23,381,765	185,318,521
Dbia.d101g	*flam*-syntenic	1.0000	0.0179	283	23,381,765	185,318,521
Dbia.d15genomes	*flam*-syntenic	0.9750	0.0143	661	2,791,184	182,453,935
Dere.GCF_003286155	*flam*-syntenic	0.7500	0.0032	94	22,146,549	146,538,397
Dere.d101g	*flam*-syntenic	0.9211	0.0002	499	20,389,455	135,657,613
Dere.droEre1	*flam*-syntenic	0.7692	0.0004	5,124	18,750,251	152,862,534
Dere.d15genomes	*flam*-syntenic	0.7500	0.0000	58	16,960,765	130,293,209
Dsuz.GCF_013340165	*flam*-syntenic	1.0000	0.0411	546	2,609,782	268,012,156
Dtei.GCF_016746235	*flam*-syntenic	0.7806	0.0000	91	24,551,875	149,494,471
Dtei.d101g_2733	*flam*-syntenic	0.9730	0.0186	831	8,218,613	152,423,477
Dtei.d101g_CT02	*flam*-syntenic	0.9773	0.0102	1,144	7,096,444	144,692,533

Table S4: Performance on genome assemblies with *flam*-like clusters. Performance for the 5 kb bin, 0.025 threshold settings is shown as TPR and FPR, respectively. Species are sorted alphabetically and multiple assemblies for the same species are sorted based on N50 as an indication of assembly quality. Results shown for N90 contigs unless otherwise specified. Detailed metrics are shown in Table S7.

Assembly	Cluster	TPR	FPR	Contigs	N50	Total size
Dfic.GCF_018152265	*flamlike1*	0.9196	0.0052	838	9,958,166	167,832,931
Dfic.d101g	*flamlike1*	0.8879	0.0049	838	9,958,166	167,832,931
Dfic.GCF_000220665	*flamlike1*	0.0000	0.0024	5,754	1,050,541	152,439,475
Dosh.d101g	*flamlike2*	1.0000	0.0034	323	2,625,980	181,013,079
Dath.GCA_008121215	*flamlike3*	0.0000	0.0017	119	52,101,127	192,660,667
Dazt.GCA_005876895	*flamlike3*	0.0000	0.0102	126	17,833,963	219,083,522
Dmir.GCF_003369915	*flamlike3*	1.0000	0.0329	104	35,263,383	287,096,000
Dper.GCF_003286085	*flamlike3*	1.0000	0.0362	432	5,212,974	195,512,972
Dper.d101g	*flamlike3*	1.0000	0.0073	1,072	4,059,666	152,810,519
Dper.d15genomes	*flamlike3*	1.0000	0.0166	415	3,429,058	163,933,157
Dpse.d15genomes	*flamlike3*	0.9796	0.0225	361	2,983,193	159,031,139
Dpse.GCF_009870125	*flamlike3*	0.8906	0.0198	70	32,422,566	163,282,969
Dinn.GCF_004354385	*flamlike4*	0.0000	0.0003	363	29,570,363	167,978,031
Damb.d101g	*flamlike5*	0.9714[1]	0.0053[1]	172	11,334,561	161,264,264
Dbif.GCA_009664405	*flamlike5*	0.7600	0.0040	214	48,071,510	192,749,618
Dobs.d101g	*flamlike5*	0.8571	0.0121	215	3,931,141	179,834,232
Dobs.GCF_018151105	*flamlike5*	1.0000	0.0079	215	3,931,141	179,834,232
Dtris.d101g	*flamlike5*	1.0000	0.0003	275	5,059,044	158,167,647

[1] Results shown for N100 contigs since *flamlike5* is located on a contig below the N90 threshold.

Table S5: Performance on genome assemblies with *flam*-syntenic clusters [5 kb bins, 0.075 threshold settings]. Performance for the 5 kb bin, 0.075 threshold settings is shown as TPR and FPR, respectively. Species are sorted alphabetically and multiple assemblies for the same species are sorted based on N50 as an indication of assembly quality. Results shown for N90 contigs unless otherwise specified. Detailed metrics are shown in Table S7.

Assembly	Cluster	TPR	FPR	Contigs	N50	Total size
Dbia.GCF_018148935	*flam*-syntenic	0.9783	0.0116	283	23,381,765	185,318,521
Dbia.d101g	*flam*-syntenic	0.9783	0.0127	283	23,381,765	185,318,521
Dbia.d15genomes	*flam*-syntenic	0.9500	0.0093	661	2,791,184	182,453,935
Dere.GCF_003286155	*flam*-syntenic	0.7500	0.0067	94	22,146,549	146,538,397
Dere.d101g	*flam*-syntenic	0.9211	0.0002	499	20,389,455	135,657,613
Dere.droEre1	*flam*-syntenic	0.6731	0.0008	5,124	18,750,251	152,862,534
Dere.d15genomes	*flam*-syntenic	0.7500	0.0005	58	16,960,765	130,293,209
Dsuz.GCF_013340165	*flam*-syntenic	1.0000	0.0449	546	2,609,782	268,012,156
Dtei.GCF_016746235	*flam*-syntenic	1.0000	0.0000	91	24,551,875	149,494,471
Dtei.d101g_2733	*flam*-syntenic	0.9730	0.0189	831	8,218,613	152,423,477
Dtei.d101g_CT02	*flam*-syntenic	0.9545	0.0077	1,144	7,096,444	144,692,533

Table S6: Performance on genome assemblies with *flam*-like clusters [5 kb bins, 0.075 threshold settings]. Performance for the 5 kb bin, 0.075 threshold settings is shown as TPR and FPR, respectively. Species are sorted alphabetically and multiple assemblies for the same species are sorted based on N50 as an indication of assembly quality. Results shown for N90 contigs unless otherwise specified. Detailed metrics are shown in Table S7.

Assembly	Cluster	TPR	FPR	Contigs	N50	Total size
Dfic.GCF_018152265	*flamlike1*	0.9018	0.0084	838	9,958,166	167,832,931
Dfic.d101g	*flamlike1*	0.8707	0.0084	838	9,958,166	167,832,931
Dfic.GCF_000220665	*flamlike1*	0.0000	0.0018	5,754	1,050,541	152,439,475
Dosh.d101g	*flamlike2*	1.0000	0.0040	323	2,625,980	181,013,079
Dath.GCA_008121215	*flamlike3*	0.9937	0.0020	119	52,101,127	192,660,667
Dazt.GCA_005876895	*flamlike3*	1.0000	0.0360	126	17,833,963	219,083,522
Dmir.GCF_003369915	*flamlike3*	0.9560	0.0715	104	35,263,383	287,096,000
Dper.GCF_003286085	*flamlike3*	1.0000	0.0433	432	5,212,974	195,512,972
Dper.d101g	*flamlike3*	1.0000	0.0093	1,072	4,059,666	152,810,519
Dper.d15genomes	*flamlike3*	1.0000	0.0202	415	3,429,058	163,933,157
Dpse.d15genomes	*flamlike3*	0.9796	0.0233	361	2,983,193	159,031,139
Dpse.GCF_009870125	*flamlike3*	0.8906	0.0427	70	32,422,566	163,282,969
Dinn.GCF_004354385	*flamlike4*	1.0000	0.0018	363	29,570,363	167,978,031
Damb.d101g	*flamlike5*	0.9714[1]	0.0116[1]	172	11,334,561	161,264,264
Dbif.GCA_009664405	*flamlike5*	0.7600	0.0036	214	48,071,510	192,749,618
Dobs.d101g	*flamlike5*	0.8571	0.0100	215	3,931,141	179,834,232
Dobs.GCF_018151105	*flamlike5*	1.0000	0.0129	215	3,931,141	179,834,232
Dtris.d101g	*flamlike5*	0.9231	0.0005	275	5,059,044	158,167,647

[1] Results shown for N100 contigs since *flamlike5* is located on a contig below the N90 threshold.

Table S7: Detailed performance for top six model settings.

- ⇨ Table available in a separate Excel file.

Table S8: proTRAC piRNA cluster predictions for *D. ficusphila*. Clusters overlapping *flamlike1* are shown with an orange background.

Chromosome	Start	End	Strand	Reads	1U fraction	10A fraction	piRNA-sized fraction
chrUn_025063985	37114	44001	+	15484	0.78	0.28	0.92
chrUn_025063993	449731	455926	-	11878	0.85	0.19	0.93
chrUn_025064091	364257	369917	+	11198	0.86	0.35	0.92
chrUn_025064091	376862	382304	+	10806	0.85	0.36	0.93
chrUn_025064091	1301599	1308329	.	14939	0.86	0.37	0.93
chrUn_025064091	1333411	1345128	+	32080	0.85	0.30	0.90
chrUn_025064091	1420415	1429405	-	15942	0.85	0.26	0.91
chrUn_025064091	1659300	1665116	-	19783	0.75	0.16	0.94
chrUn_025064091	1729905	1737103	+	17625	0.83	0.28	0.93
chrUn_025064091	1793131	1799557	+	8639	0.85	0.16	0.90
chrUn_025064092	1314113	1319927	-	8224	0.87	0.33	0.95
chrUn_025064092	9849861	9856888	+	19588	0.78	0.38	0.91
chrUn_025064092	13781325	13789616	-	28620	0.83	0.26	0.92
chrUn_025064092	13969206	13975554	-	15898	0.84	0.15	0.93
chrUn_025064092	14948810	14954280	.	7352	0.84	0.27	0.94
chrUn_025064094	1457100	1466214	+	15920	0.82	0.19	0.93
chrUn_025064118	480411	495894	+	32320	0.83	0.35	0.93
chrUn_025064156	1099601	1104901	-	7771	0.92	0.26	0.93
chrUn_025064203	330004	337808	+	19705	0.81	0.37	0.93
chrUn_025064220	20507	27607	-	17042	0.84	0.13	0.95
chrUn_025064220	47507	54222	+	10114	0.85	0.46	0.92
chrUn_025064328	13150806	13155969	-	12001	0.81	0.36	0.90
chrUn_025064328	20922251	20927720	+	14071	0.86	0.17	0.94
chrUn_025064328	21103451	21110345	-	12608	0.87	0.23	0.93
chrUn_025064328	21822401	21829520	-	13708	0.84	0.24	0.93
chrUn_025064328	22182915	22187919	+	7222	0.79	0.31	0.91
chrUn_025064328	22616744	22627212	-	25681	0.82	0.15	0.93
chrUn_025064328	23480546	23487171	-	14788	0.83	0.38	0.92
chrUn_025064328	24848015	24853713	+	9943	0.76	0.37	0.90
chrUn_025064347	4711	11292	+	19264	0.87	0.29	0.91
chrUn_025064389	3256519	3263718	+	16902	0.77	0.27	0.92
chrUn_025064569	474985	480619	.	8077	0.88	0.37	0.93
chrUn_025064569	518301	526775	-	16148	0.86	0.28	0.92
chrUn_025064569	586919	596619	-	19150	0.83	0.27	0.86
chrUn_025064569	623006	630719	-	17978	0.91	0.22	0.92
chrUn_025064569	631712	640974	-	27551	0.90	0.22	0.92
chrUn_025064569	691620	697110	.	8271	0.85	0.28	0.90
chrUn_025064569	707805	712826	-	9765	0.88	0.28	0.94
chrUn_025064569	736060	742494	-	21302	0.88	0.28	0.94
chrUn_025064569	751914	763424	-	60312	0.91	0.31	0.93
chrUn_025064569	846513	852303	.	12365	0.90	0.50	0.89

chrUn_025064569	853629	866914	-	117939	0.92	0.30	0.91
chrUn_025064569	870849	878823	-	74824	0.89	0.28	0.91
chrUn_025064569	879435	1006512	-	1836023	0.90	0.25	0.92
chrUn_025064569	1011837	1021318	-	34302	0.87	0.26	0.92
chrUn_025064569	1021601	1039775	-	122068	0.87	0.31	0.93
chrUn_025064569	2019339	2024510	-	6549	0.88	0.39	0.93
chrUn_025064569	2438005	2445385	+	16752	0.76	0.27	0.92
chrUn_025064719	1	5065	-	18498	0.89	0.19	0.94
chrUn_025064737	5265869	5272074	-	10639	0.82	0.39	0.90

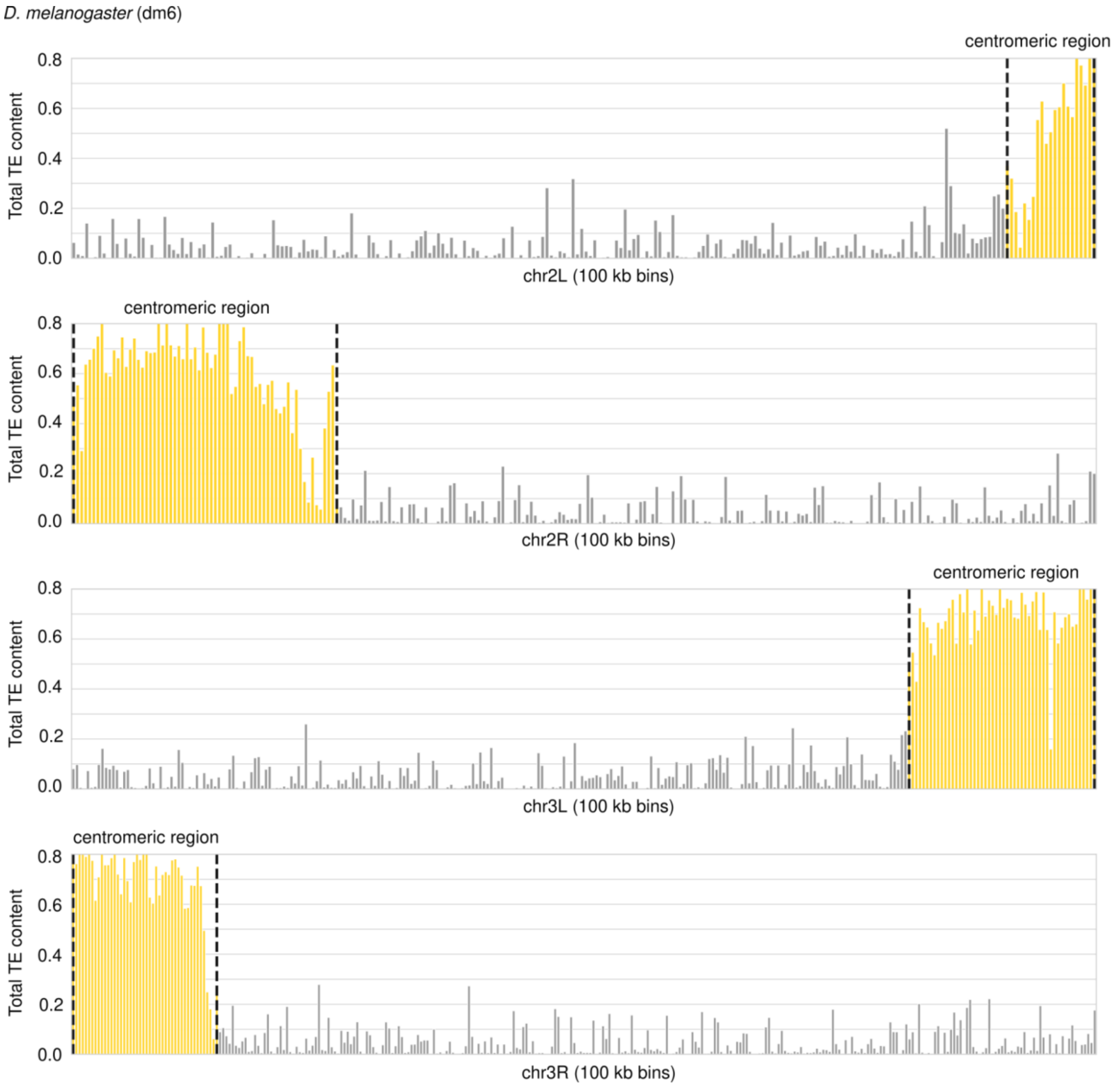

Figure S1: Automatic annotation of centromeric regions. Total TE content is shown across 100 kb bins on the left (L) and right (R) arm of chr2 and chr3 in *D. melanogaster*. A sliding window of five 100 kb bins was used to evaluate the TE content and centromeric regions were defined to start at the chromosome end and extend for as long as the TE content across the 5 bins was above 20 %. Centromeric regions are shown in yellow within the black dashed lines.

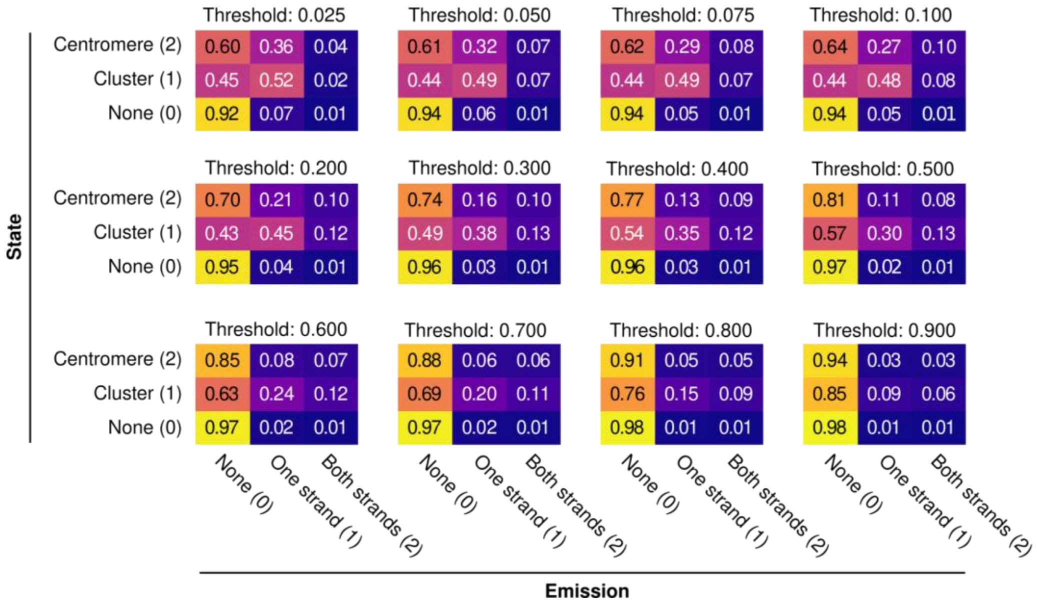

Figure S2: Emission matrices depend on TE content threshold. Emission matrices for *D. melanogaster* across different Gypsy content thresholds and using the 5 kb binning strategy. Emissions were defined based on the predicted LTR Gypsy content in each bin and categorised as: no Gypsy elements (None; 0), Gypsy elements present on one strand (One strand; 1), Gypsy elements on both strands (Both strands; 2).

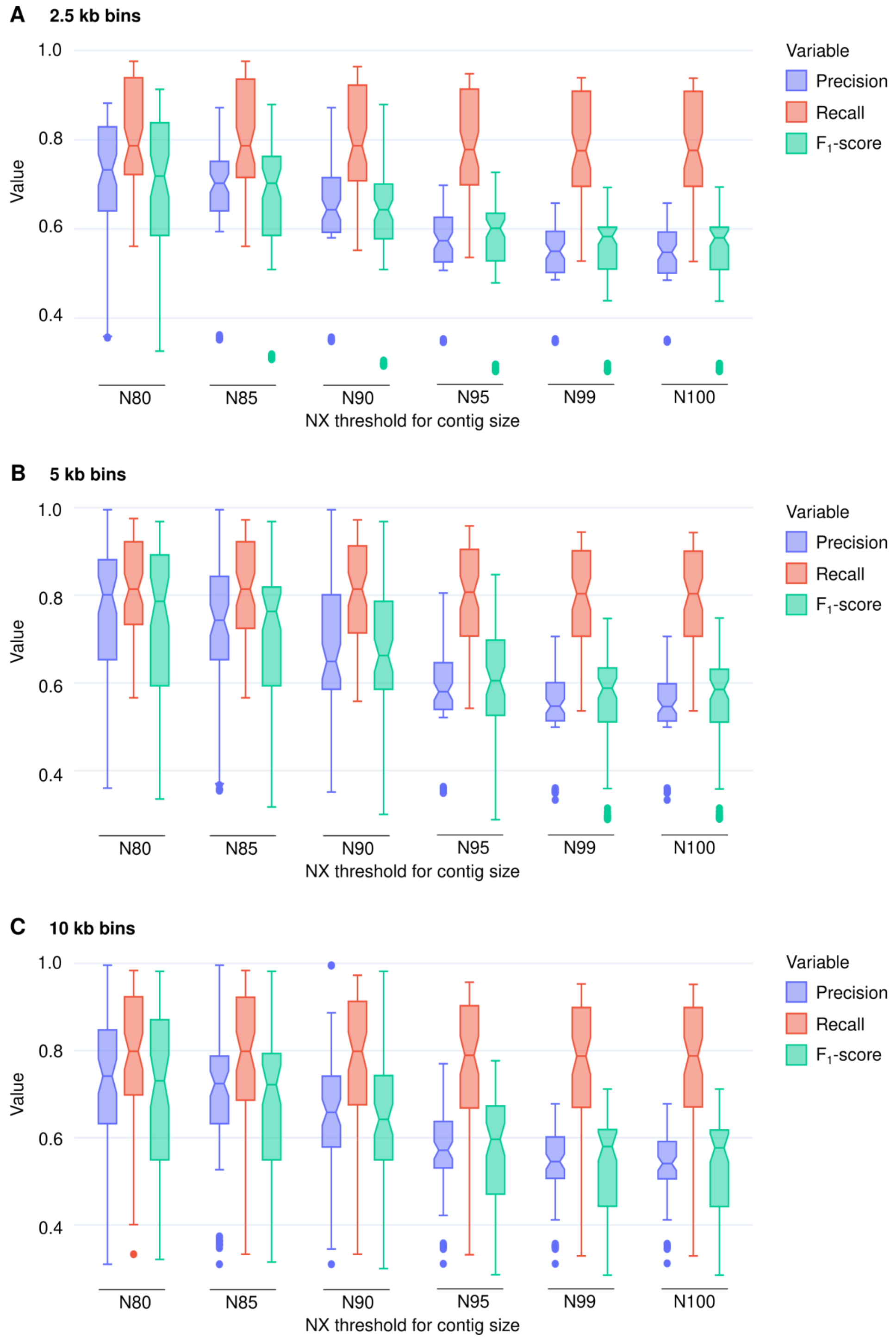

Figure S3: Overview of model performance by NX threshold and bin size. Cross validation model performance (mean across all three classes and all tested emission thresholds) using six different NX metric thresholds across (A) 2.5 kb, (B) 5 kb, or (C) 10 kb bins. Please note that N90 corresponds to the results presented throughout the study.

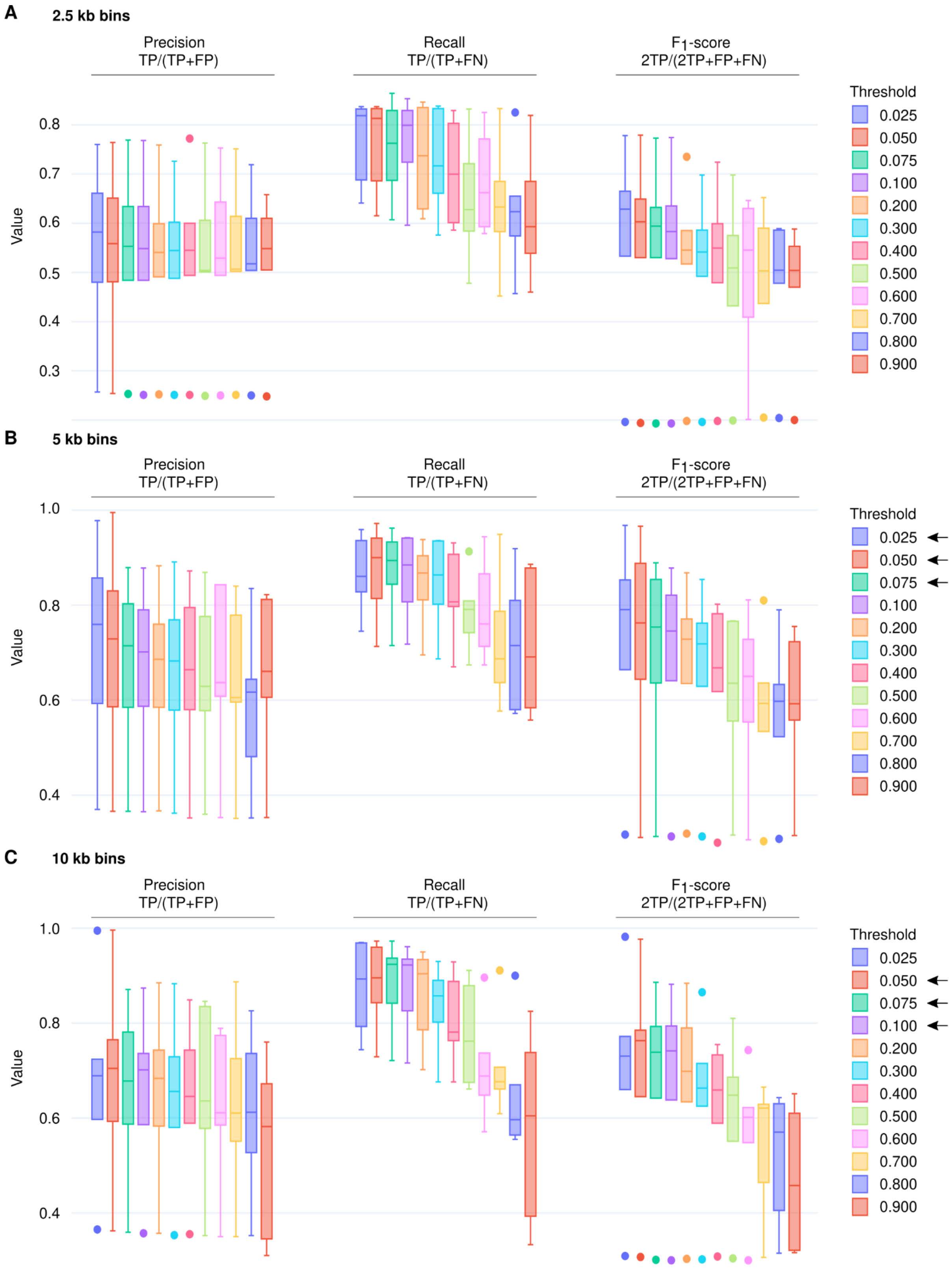

Figure S4: Overview of model performance by threshold and bin size. Cross validation model performance (mean across all three classes) using three evaluation metrics for 12 different thresholds for emission calculation across (A) 2.5 kb, (B) 5 kb, or (C) 10 kb bins. The thresholds with best F_1-scores are indicated with arrows.

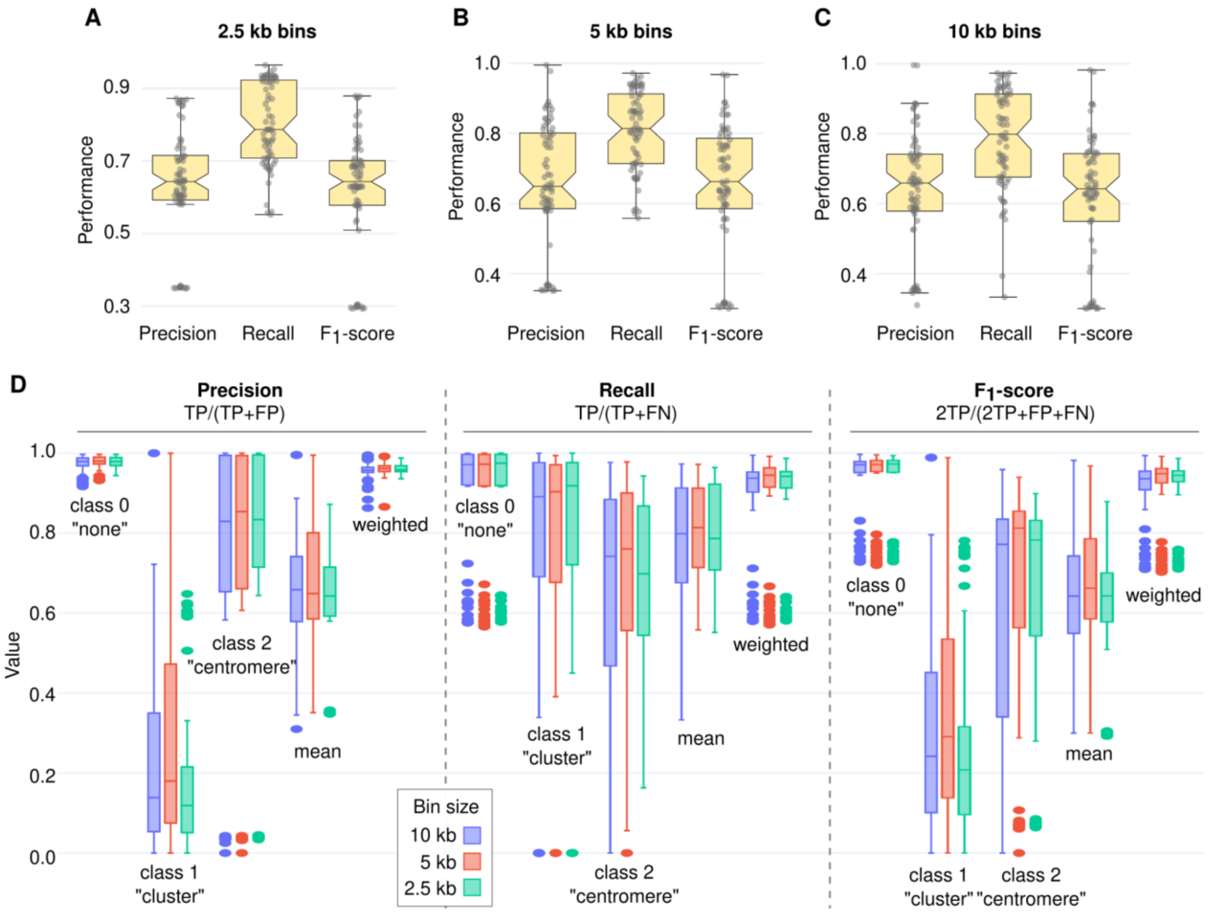

Figure S5: Additional details on model performance. Overall model performance based on cross validation using either (A) 2.5 kb, (B) 5 kb, or (C) 10 kb bins. (D) Detailed model performance with evaluation metrics for each class of the model (None, Cluster and Centromere), or the arithmetic or weighted mean using 10 kb, 5 kb, or 2.5 kb bins. All results are shown across all investigated emission thresholds.

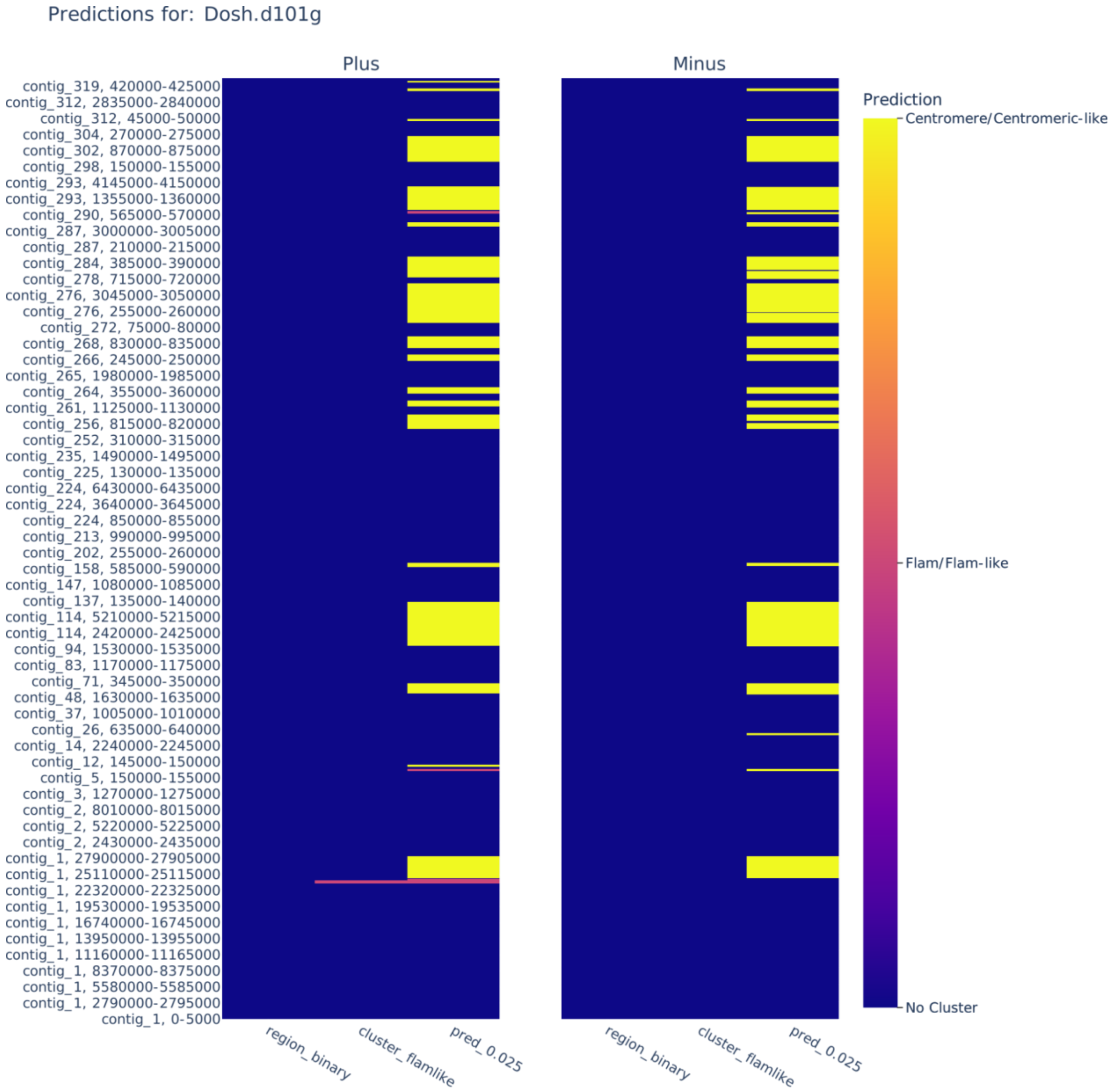

Figure S6: Model output for *D. oshimai*. Example of cluster prediction and visualisation for *D. oshimai* using bin size 5 kb and TE threshold 0.025. Annotations are shown as centromere (yellow), *flam*-like (pink), and none (blue) across N90 contigs.

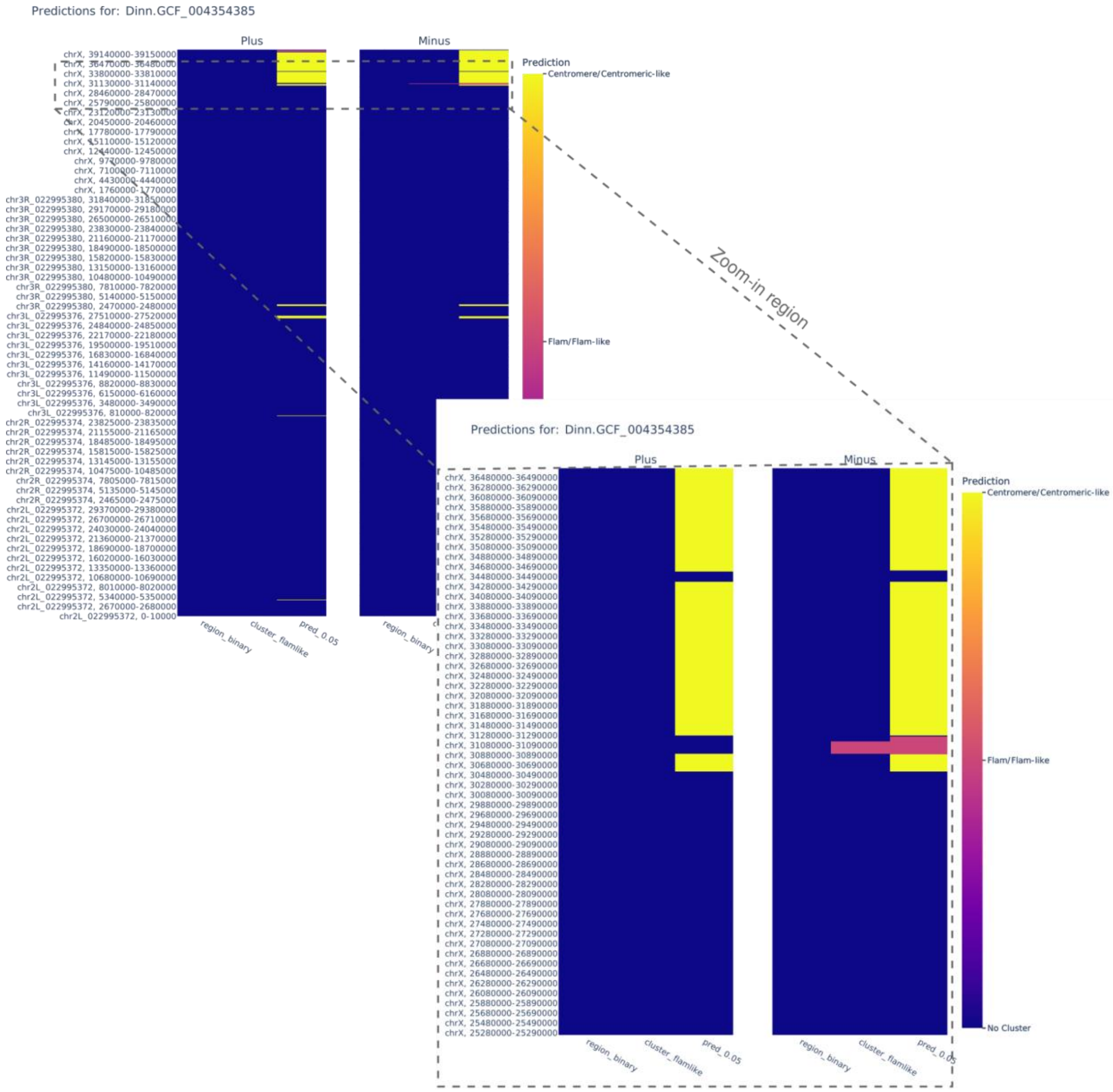

Figure S7: Model output for *D. innubila*. Example of cluster prediction and visualisation for *D. innubila* using bin size 10 kb and threshold 0.05. The visualisation allows the user to zoom interactively. An example zoom-in region is shown at the bottom right. Annotations are shown as centromere (yellow), *flam*-like (pink), and none (blue) across N90 contigs.

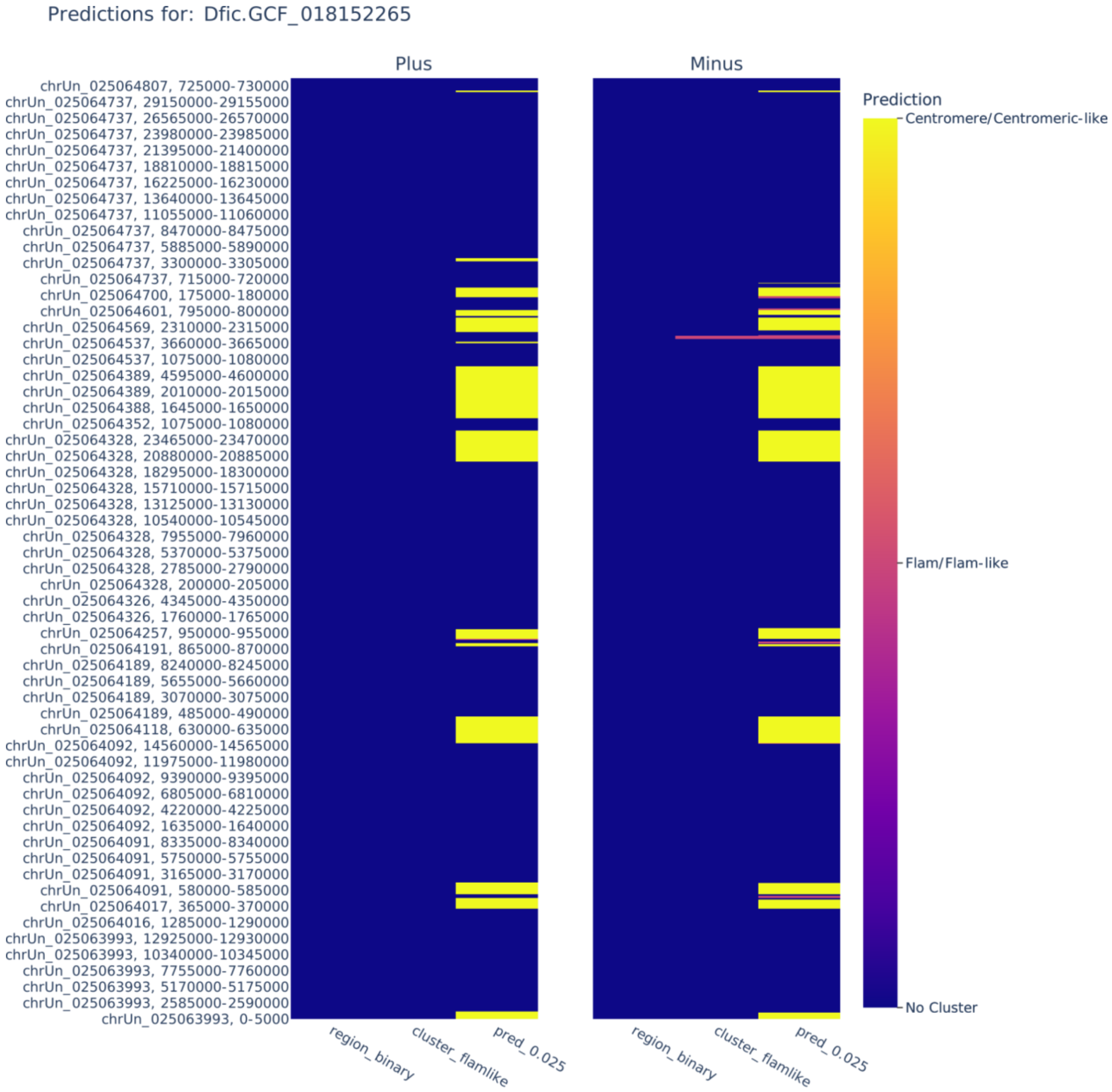

Figure S8: Model output for *D. ficusphila*. Example of cluster prediction and visualisation for *D. ficusphila* using bin size 5 kb and threshold 0.025. Annotations are shown as centromere (yellow), *flam*-like (pink), and none (blue) across N90 contigs.

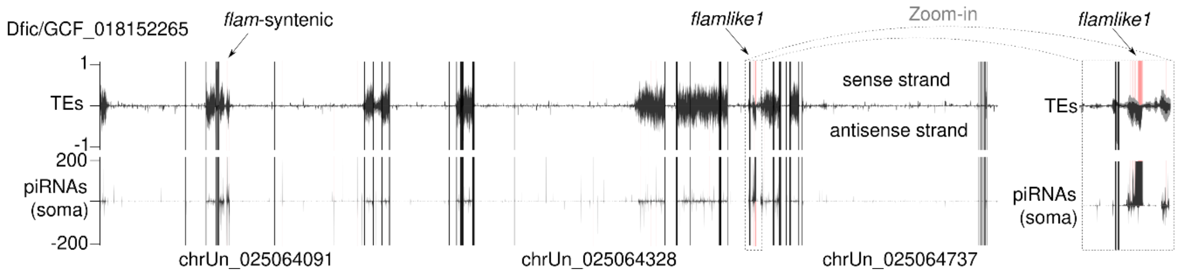

Figure S9: Overview of proTRAC predictions for *D. ficusphila*. proTRAC piRNA cluster predictions across the *D. ficusphila* genome. Genomic coordinates are shown on the x axis with vertical black lines indicating contig breaks and selected contig names indicated. The top tracks show transposon content (black lines, LTR TEs; grey lines, all TEs) and the bottom one (black lines) shows piRNAs in soma-enriched ovarian cells (4). Positive values represent the sense strand and negative values the antisense strand relative to *flamlike1*. proTRAC predictions are shown as shaded pink areas. The major piRNA cluster predicted corresponds to previously reported *flamlike1* (4), indicated by an arrow and shown in the zoom-in to the right (grey dashed lines).

References

1. Chang,C.H., Chavan,A., Palladino,J., Wei,X., Martins,N.M.C., Santinello,B., Chen,C.C., Erceg,J., Beliveau,B.J., Wu,C.T., *et al.* (2019) Islands of retroelements are major components of Drosophila centromeres. *PLoS Biol*, **17**.
2. Talbert,P.B., Kasinathan,S. and Henikoff,S. (2018) Simple and Complex Centromeric Satellites in Drosophila Sibling Species. *Genetics*, **208**, 977–990.
3. Klein,S.J. and O'Neill,R.J. (2018) Transposable elements: genome innovation, chromosome diversity, and centromere conflict. *Chromosome Res*, **26**, 5–23.
4. van Lopik,J., Alizada,A., Trapotsi,M.A., Hannon,G.J., Bornelöv,S. and Czech Nicholson,B. (2023) Unistrand piRNA clusters are an evolutionarily conserved mechanism to suppress endogenous retroviruses across the Drosophila genus. *Nat Commun*, **14**, 7337.
